# Effects of Lysozyme on the Activity of Ionic of Fluoroquinolone Species

**DOI:** 10.3390/molecules23040741

**Published:** 2018-03-23

**Authors:** Hugo Alejandro Perez, Ana Yanina Bustos, María Pía Taranto, María de los Angeles Frías, Ana Estela Ledesma

**Affiliations:** 1 Departamento de Química, Facultad de Ciencias Exactas y Tecnologías, Universidad Nacional de Santiago del Estero-CONICET, Av. Belgrano (S) No. 1912, 4200 Santiago del Estero, Argentina; magnihap@hotmail.com; 2 Laboratorio de Biointerfases y Sistemas Biomimeticos, Centro de Investigación en Biofísica Aplicada y Alimentos (CIBAAL), Universidad Nacional de Santiago del Estero—CONICET, RN 9, Km 1125, 4206 Santiago del Estero, Argentina; marafrias@hotmail.com; 3 Centro de Investigación en Biofísica, Aplicada y Alimentos (CIBAAL), Universidad Nacional de Santiago del Estero—CONICET, RN 9, Km 1125, 4206 Santiago del Estero, Argentina; yanina_bioq04@hotmail.com; 4 Facultad de Ciencias Médicas, Universidad Nacional de Santiago del Estero-CONICET, Av. Belgrano (S) No. 1912, 4200 Santiago del Estero, Argentina; 5 Centro de Referencia para Lactobacilos (CERELA-CONICET), Chacabuco 145, 4000 San Miguel de Tucumán, Argentina; ptaranto@cerela.org.ar

**Keywords:** fluoroquinolone, lysozyme, DFT calculations, docking, antimicrobial activity

## Abstract

Fluoroquinolones (FQs) constitute an important class of biologically active broad-spectrum antibacterial drugs that are which are in contact with many biological fluids under different acidity conditions. We studied the reactivity of ciprofloxacin (Cpx) and levofloxacin (Lev) and their interaction with lysozyme (Lyz) at different pH values, using UV-visible absorption, fluorescence, infrared spectroscopies supported by DFT calculation and docking. In addition, by antimicrobial assays, the biological consequences of the interaction were evaluated. DFT calculation predicted that the FQ cationic species present at acid pH have lower stabilization energies, with an electric charge rearrangement because of their interactions with solvent molecules. NBO and frontier orbital calculations evidenced the role of two charged centers, NH_2_^+^ and COO^−^, for interactions by electronic delocalization effects. Both FQs bind to Lyz via a static quenching with a higher interaction in neutral medium. The interaction induces a structural rearrangement in β-sheet content while in basic pH a protective effect against the denaturation of Lyz was inferred. The analysis of thermodynamic parameters and docking showed that hydrophobic, electrostatic forces and hydrogen bond are the responsible of Cpx-Lyz and Lev-Lyz associations. Antimicrobial assays evidenced an antagonist effect of Lyz in acid medium while in neutral medium the FQs’ activities were not modified by Lyz.

## 1. Introduction

**Figure 1:**
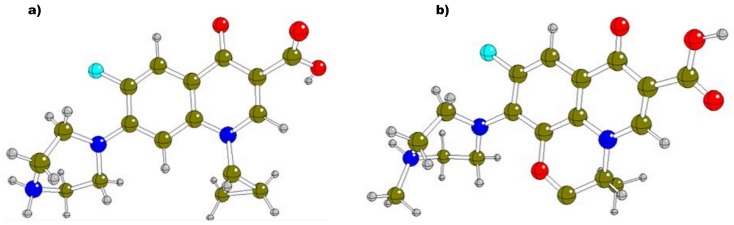
Chemical structures of Ciprofloxacin (Cpx) (**a**) and Levofloxacin (Lev) (**b**).

Fluoroquinolones (FQs) are synthetic antibiotics with a broad antimicrobial spectrum that includes Gram-positive and Gram-negative bacteria [1,2]. The pharmacological activity of these compounds is due to the fact that they can act at the level of bacterial DNA replication by inhibiting the enzyme topoisomerase IV or DNA gyrase [3,4,5,6]. These compounds result from the addition of a fluorine atom at the 6-position and a piperazine ring at carbon 7 to their predecessor, nalidixic acid [7]. These substituents give rise to new structures such as ciprofloxacin (Cpx) and levofloxacin (Lev), which show improvements in both biological activity and pharmacokinetic properties [8,9,10,11] (Figure 1). Cpx is the most representative FQ, with the highest pharmacological use of this compound family. Lev is structurally different from Cpx due to the presence of an oxacin ring in the 8-position of the quinolone group that provides a wider antibacterial coverage at a reduced dosage although absorption is delayed in the presence of food [12].

It is well known that the physicochemical properties of FQs are strongly affected by pH changes according to Fasani et al. [13]. These compounds often have different water solubility, reactivity with chemical oxidants and UV absorption. In the last decade, several works have reported studies on the acid-base equilibrium of FQs, in which potentiometric titration, conductimetry and spectrophotometry were used [14,15,16]. Previous results showed that Cpx species form different types of complexes as a function of pH in aqueous solution [17].

On the one hand, the comparative performance of different DFT methods and different basis sets in the calculations of the molecular structure and vibrational spectra of Cpx have been investigated [18]. On the other hand, properties of Lev using DFT/B3LYP method were reported only for neutral species containing COOH and N-CH_3_ groups [19].

When administered, FQs can interact with proteins such as Lyz and therefore their antimicrobial activity could be affected. Lysozyme (Lyz) is a protein that contains 129 amino acid residues, with four disulfide bonds, six tryptophane (Trp), three tyrosine (Tyr) and three phenylalanine (Phe) residues [20], present in various biological fluids and tissues, including skin, saliva, tears, liver blood and lymphatic tissues of humans and animals [20,21,22]. 

Recent reports have characterized the interaction between FQs and Lyz by different spectroscopic studies [20,23,24]. Our investigation is important because, to our knowledge there are no available studies about the interactions of different FQs ionic species with proteins and their effects on the antimicrobial activity. Normally, the species present in solution can adopt three forms, namely, zwitterionic, anionic and cationic, depending on the pH of aqueous solution, so the purpose of this research is the study of the binding between FQs ionic species and Lyz protein and its effects on the antimicrobial activity. Experimental techniques, including in situ ATR-FTIR, fluorescence and antimicrobial assays were employed to gain new insights into this issue. All of them were supported by DFT calculations and molecular docking studies.

## 2. Results and Discussion

### 2.1. Optimization of FQs Species

In Table 1 the stabilization energy (E) and relative energies (ΔE), dipole moments (μ(D)) and solvation energies (ΔG_solv_) for Cpx and Lev species calculated at pH 4.5, 7.4 and 10 in aqueous solution using the B3LYP/LANL2DZ method are listed. The E parameter shows that all ionic species of Lev are more stable than Cpx ones, taking into account their absolute values. From the analysis of E, we infer that the protonation of the amine group in acid medium gives more electronic stabilization to the FQs. The stabilization energy differences of 1206 kJ/mol for zwitterionic and 2413 kJ/mol for anionic Cpx structures with values similar to the corresponding Lev species suggest that the protonation of the amine group brings on an electronic stabilization of the FQs molecules. These theoretical results agree with previous experimental reports [24].

The free energy of solvation is an important parameter to evaluate the stability in solution, indicating the changes in free energies of the solute due to a dissolution process. In this thermodynamic parameter, the corrected solvation energy (ΔG_solv_) is defined as the thermal contribution to the Gibbs free energy and nonelectrostatic component due to the cavitation, dispersion and repulsion energies [25]. The value obtained for ΔG_solv_ is greater for Cpx zwitterionic specie (−440.7 kJ/mol) than for the remaining cationic and anionic species. Lev showed the same behavior. Furthermore, ΔG_solv_ values for Lev are more negative than those expected for Cpx, resulting from the presence of the oxacin ring. The aqueous medium confers more stabilization energy, especially for the zwitterionic species of Lev antibiotic. The higher dipole moment (μ) values of 61.9 D for Cpx and 58.6 D for Lev observed for zwitterionic system favors the antibiotic-water interaction. These values are in full agreement with the data obtained for ΔG_solv_.

### 2.2. Theoretical Stabilities and Reactivities of FQs in Aqueous Medium

We evaluated the stabilization of FQs analyzing the frontier orbitals. Our GAP values (energy difference between HOMO and LUMO orbitals) calculated at the B3LYP/LANL2DZ level of theory show that Lev species presents a lower GAP value than Cpx in the three studied media, being the most reactive species with a GAP value of −3.2658 eV (Table S1). On the basis of Koopmans’ theorem [26], the reactivity and site selectivity of a molecular system can be describe using electronic descriptors such as chemical potential (*μ*). The negative chemical potential values observed for all molecules especially at pH 4.5, indicate a major structural stability of FQs cationic species.

The donor-acceptor energy interactions are important parameters that can predict the stabilities of FQs due to the presence of O and N atoms with lone pairs. Hence, the charge transfers were investigated with Natural Bond Orbital (NBO) analysis. In Table S2 the NBO results for anionic, zwitterionic and cationic species are analyzed. It is clearly observed that the zwitterionic species shows the highest total delocalization energy values, and, for this reason, it presents higher stability in water than the remaining species (Figure S1). Hence, in neutral medium, the presence of the two charged centers such as NH_2_^+^ and COO^−^ increases the stability of zwitterionic species by electronic delocalization. This analysis is in good agreement with the above results.

### 2.3. Spectral Differences of Anionic, Zwitterionic and Cationic FQs Species 

**Figure 2:**
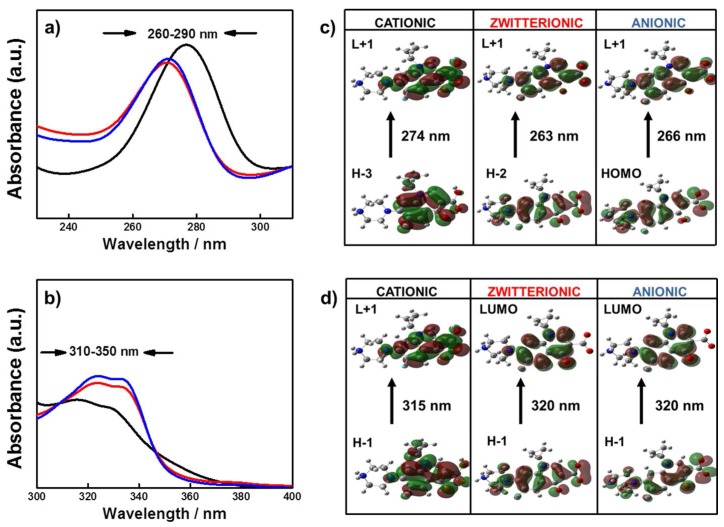
(**a**) Absorption spectra of Cpx in the region of 220–310 nm and (**b**) in the region from 300 to 400 nm at pH 4.5 (black solid line), pH 7.4 (red solid line) and pH 10 (blue solid line). (**c**,**d**) Selected molecular orbital plots corresponding to orbital-orbital excitations of Cpx obtained by the B3LYP/LANL2DZ method.

It is known that the UV-Vis spectra for Cpx and Lev show two bands, an intense one at 260–280 nm and another lower intensity one in the 320–340 nm region [1]. Figure 2a shows a hypsochromic shift (lower wavelength around 6–7 nm) for Cpx at the first transition and a bathochromic shift (towards higher wavelength) for the second band (Figure 2b) with the increase of pH values.

For a better interpretation of the experimental band shift, we performed theoretical calculations. For the first band of Cpx experimentally determined, theoretical calculations predict the main transition from the H−3 orbital to the first excited state L+1 which is π → π* in nature (Figure 2c). This transition implies a homogenous electron density transfer to all surface molecules for zwitterionic and anionic species while for cationic ones, this transition excludes the piperazine ring. The second excitation is originated from H−1 to L+1 orbitals for cationic, H−1 to LUMO for zwitterionic and H−1 to LUMO for anionic species with π → π* transitions (Figure 2d). These results explain the band shifts experimentally observed at each pH. A similar behavior was observed for Lev (data not shown).

### 2.4. FTIR Spectra of FQs Species

**Figure 3:**
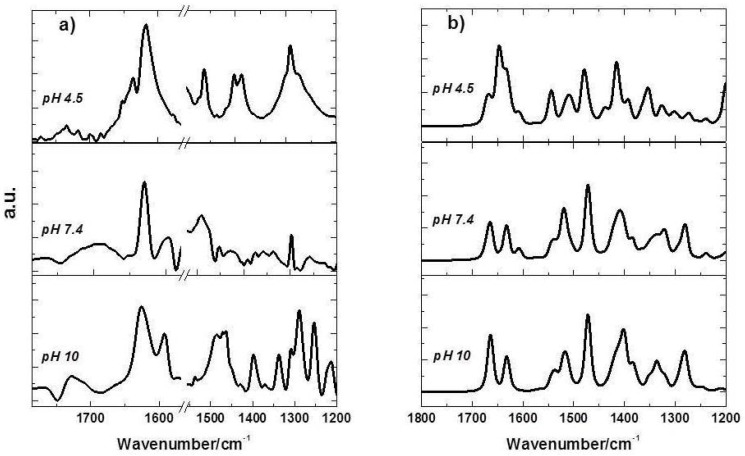
(**a**) ATR-FTIR experimental spectra of Cpx solution and (**b**) theoretical spectra at pH 4.5 (**upper panel**), pH 7.4 (**middle panel**) and 10 (**bottom panel**).

Vibrational spectroscopy is one of the few methods that directly report on the strength of hydrogen bonds that can stabilize the structure of a molecule. The principal absorption bands for FQs using ATR-FTIR spectroscopy were assigned taking into account the theoretical vibrational modes obtained as described in Section 3. As a general rule, hydrogen bonding lowers the frequency of stretching vibrations, since it lowers the restoring force, but increases the frequency of bending vibrations since it produces an additional restoring force [27]. In Figure 3a the spectra of Cpx and Lev in neutral medium do not show the presence of the *ν*_COOH_ vibration as was established by X-ray diffraction [28]. In basic medium, Cpx shows a characteristic band of the symmetric stretching of the deprotonated carboxylate group at 1397.0 cm^−1^ while in the acid medium a shift to lower wavenumber is observed (1387.0 cm^−1^) indicating the interaction of Cpx with the medium by the formation of hydrogen bonds. In the case of Lev the same shift to lower frequencies from the basic to acid medium was observed. In the same Figure 3a, when the pH is greater than pK_a1_ (6.27) [29,30] the deprotonation of the carboxyl group reduces the intensity of coupled COOH and a weak band is observable at approximately 1257.3 cm^−1^, as was reported by Yan et al. and references therein [31]. The band corresponding to the asymmetric vibration of COO^−^ appears at 1593.5 cm^−1^ at pH 10 and 1595 cm^−1^ at pH 7.4, but it is not observable at pH 4.5. Another important peak to analyze is the ketone C=O stretch (υ_C=O_); whole peak position shifts from 1634.0 cm^−1^ at pH 4.5 to 1624.0 cm^−1^ at pH 10. The correct identification of C-N stretching frequency is not very easy because in that area a mixture of bands can be found. The IR bands observed at 1538.0 and 1507.0 cm^−1^ have been assigned to C-N symmetric and asymmetric stretching vibrations, in good agreement with those reported by Gunasekaran et al. [32,33]. All the data mentioned before are in good correlation with the vibrational spectra calculated at the B3LYP/LANL2DZ level of theory, as was shown in Figure 3b.

### 2.5. Effects of FQs Molecules on the Structure of Lyz by FTIR Spectroscopy

Infrared spectroscopy is an extremely useful tool for the investigation of protein structure since it provides information on the structure and environment of the protein backbone and of the amino acid side chains [34]. 

**Figure 4:**
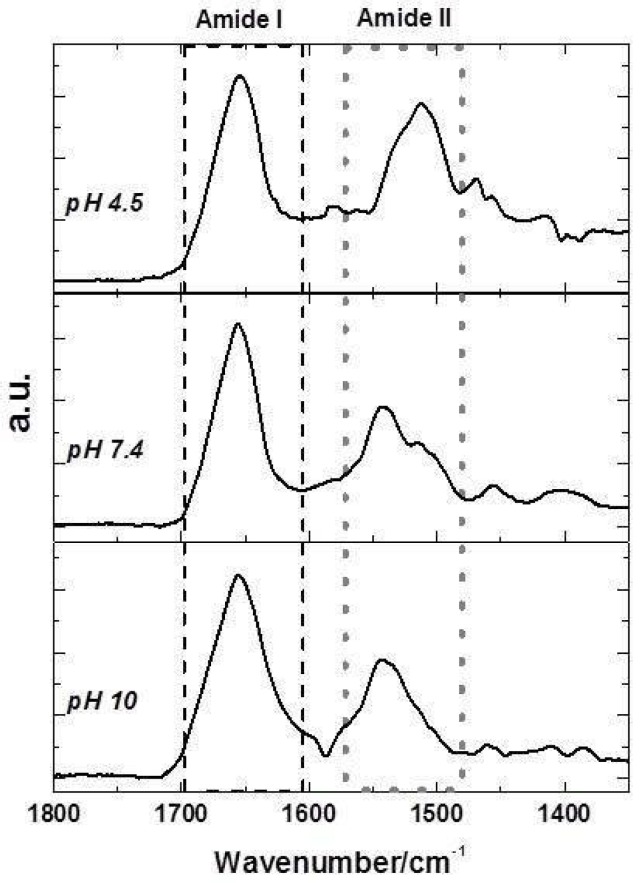
FTIR spectra of Lyz protein in the range between 1800 and 1400 cm^−1^ at pH 4.5 (**upper panel**), pH 7.4 (**middle panel**) and pH 10 (**bottom panel**).

One advantage of infrared spectroscopy is that the protein backbone as well as the side chains can be observed in the same experiment, providing quantitative information. It is usually accepted that CD measurements provide more accurate estimations of the protein α-helix content, whereas IR is thought to be more sensitive to β-sheets [35]. Lyz protein structural changes could be analyzed using the experimental FTIR spectrum. The intense broad band centered at 1655.0 cm^−1^ is attributed to the amide I signal, while the band at 1540.0 cm^−1^ corresponds to the amide II band. In Figure 4 the ATR-FTIR spectra of Lyz at different pH values in the range between 1800.0 and 1400.0 cm^−1^ are analyzed. The amide bands give rise to overlapping bands so a combination of Fourier deconvolution and curve-fitting procedures was used to obtain the positions and contributions of the component bands. In Table 2, the percentage of each Lyz secondary structure content at pH 4.5, 7.4 and 10 in the absence and in the presence of Cpx and Lev is presented.

The band localized at 1606.0 cm^−1^ assigned to the extended chains would indicate an interaction of the protein with the reaction medium itself (TRIS solution) [36]. The band centered at 1655 cm^−1^ corresponding to amide I was analyzed to determine the contribution of each component at the secondary structure.

At neutral pH, the secondary structure analysis of Lyz suggests that it contained 31% α-helix, 5% β-sheet, 24% turn and 30% unordered coil with a similar pattern to previously reported ones [24]. The presence of zwitterionic Cpx species induces important structural changes in Lyz, a decrease in α-helix structures with an increase in extended chain and β-sheet being observed. By examining selected literature data, it can be observed that discrepancies exist in the results. Using circular dichroism (CD) techniques some authors have demonstrated a gradual decrease of α-helix and β-sheet content of Lyz upon Cpx binding in aqueous solution [24] while others have reported an increase in α-helix content [20] in that condition. Our FTIR analysis of the Lyz structure by the interaction with zwitterionic species agrees with the decrease of α-helix content but we observed an increase of the β-sheet content of protein. Probably, the differences observed in β-sheet could be due to the greater sensitivity of FTIR spectroscopy to β-sheets compared with CD. So, these results suggest a certain degree of protein unfolding and loss of activity, which correlated well with the decrease in α-helix content [22]. Contrary to the increase in α-helix content of the Lyz-Lev system reported at this pH by Fang et al. [23], we observed a decrease of α-helix and β-turn contents. The comparison with those authors suggests that their initial Lyz secondary structure, is different from that normally reported by Pasban Ziyarat et al. [24] for Lyz in buffer pH 7.4. However, our final secondary structure of Lyz upper Lev binding is in agreement with them, so we inferred that it results in the unfolding of the protein.

At acid pH, the quantification of the secondary structure composition from the amide I band resulted in a revealed α-helix contribution of 29% and a β-sheet contribution of 12% for free Lyz, in agreement with the secondary structure content previously reported [37]. The structure of Lyz in this medium was slightly perturbed by the interaction with Cpx and Lev antibiotics, observing that the α-helix decreased gradually, from 29% in free Lyz to 27% upon Cpx binding, and it was conserved in the Lev binding. These results would indicate no important interaction of this FQs cationic species with the protein [38]. At basic pH, as a consequence of the medium, the α-helix content of Lyz decreases, accompanied by an increment in antiparallel β-sheet content and a new contribution at the secondary structure corresponding to parallel β-sheet (1690 cm^−1^) can be observed. The content of the α-helix structure of Lyz increases in the presence of the antibiotics Cpx and Lev, while the presence of Cpx anionic species slightly preserves the parallel β-sheet formation that is characteristic of fibrillar structures. This analysis shows that ionic species of Cpx and Lev to bound Lyz to trigger a conformational change in the protein, with a loss of α-helix stability in acid and neutral medium and a gain of this structure in the basic media.

### 2.6. Fluorescence Emission of FQs in Presence of Lyz

**Figure 5:**
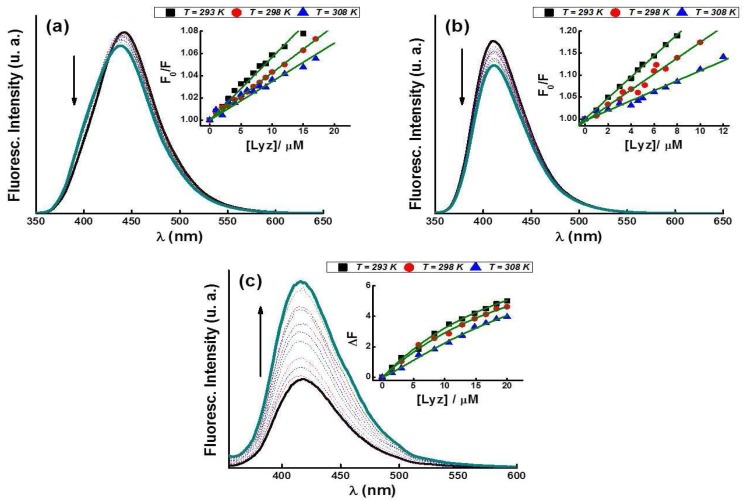
Fluorescence emission spectra of Cpx (**a**) at pH 4.5, (**b**) at pH 7.4, and (**c**) at pH 10, in the presence of different Lyz concentrations at 298 K and λ_exc_ = 330 nm. Inset Figures: Stern-Volmer plots for the quenching of Cpx by Lyz (**a**–**c**) Hill plots, at three different temperatures.

Fluorescence spectroscopy can be regarded as a widely used technique to explore the mechanism of interactions between the ligands and proteins [39,40]. In our system, both the FQs and Lyz protein are fluorescent molecules. The fluorescence spectral properties of FQs species were used to obtain detailed information on the nature of FQ-Lyz interaction. The emission spectra of Cpx upon the addition of Lyz are illustrated in Figure 5. In acid and neutral media, the fluorescence intensities of Cpx species decrease with increasing Lyz concentrations (Figure 5a,b) while at pH 10 an opposite behavior is observed (Figure 5c).

The results obtained at pH 4.5 and 7.4 can be analyzed by a quenching mechanism that can be classified as dynamic quenching. They may be distinguished by their different dependence on temperature and viscosity or, preferably, by lifetime measurements [40,41,42,43]. For dynamic quenching, the effective collision times between molecules, the energy transfer efficiency and the fluorescence quenching constants increase when the temperature of the system rises. In contrast, the static quenching constants decrease when the temperature increase resulting in a less complexes stability; thus, the values are expected to be smaller. To confirm the nature of the quenching mechanism induced by Lyz in acid and neutral media, we analyzed the graphs plotted for *F*_0_*/F* versus [Q] according to the Stern–Volmer (SV) Equation (1) at different temperatures (insets in Figure 5a,b):

(1) $$F_{0}/F=1+K_{SV}\times \left[Q\right]=1+k_{q}\times \tau_{0}\times \left[Q\right]$$

where *F*_0_ and *F* denote the steady-state fluorescence intensities in the absence and presence of quencher, respectively. *k_q_* is the quenching rate constant of the biological macromolecule; *K_SV_* is the Stern-Volmer quenching constant, [Q] is the concentration of quencher, τ_0_ is the average lifetime of the molecule without any quencher and the fluorescence lifetime of the biopolymer is 10^−8^ s [42].

In the insets of Figure 5a,b, a decrease in the slope of the equation (*K_SV_*) is observed when the temperature increases. This means that the quenching of FQs molecules by Lyz was not dynamic but static, so *K_SV_* can be interpreted as the association constant or binding constant (*K*). The calculated *K* values at pH 4.5 and 7.4 versus temperature are listed in Table 3. In addition, assuming τ_0_ = 1.10^−9^ s for the FQs [1], our calculated quenching constant *k_q_* is far greater than the *k_q_* of the diffusional constant (2.0 × 10^10^ L mol^−1^ s^−1^) [39] which confirms also the static quenching in the interaction between FQs and Lyz.

Another important result is observed at pH 10 where the fluorescence intensity of FQs increases with the increase in Lyz concentration, showing a different behavior compared to the remaining pH values. This phenomenon may be related to changes in the secondary structure of the protein at pH 10, as is shown using FTIR analysis in previous section. These spectral changes observed are analyzed using Hill Equation (2) and the adjustment of the data is shown in the inset of Figure 5c:

(2) $$\Delta F=F-F_{0}=\frac{a\times \left(F_{\infty }-F_{0}\right)\times ){\left(K\times \left[Q\right]\right)}^{n}}{1+{\left(K\times \left[Q\right]\right)}^{n}}$$

where *F*_0_ is the initial fluorescence, “*a*” is a constant of proportionality, the term *F − F*_0_ can approximate the number of FQ molecules to associate with Lyz, *F_∞_* is the fluorescence observed when all FQs molecules are completely linked to the Lyz, “*n*” is the number of binding sites and *K* is the association constant already reported in Table 3. At pH 10 the *K* values are higher than those obtained in the other two pH media. This could be explained considering that at pH 10, the FQs have a negative net charge while the Lyz protein has a positive net charge (pI = 11.5), favoring the electrostatic attraction between them.

In our work, we find that depending on the ionic state of the FQs, which is dependent on the local pH of the immediate environment, the compound may interact with different affinity with relevant proteins such as Lyz present around. The λ_exc_ used in our work is equal to 330 nm where only the FQ species are excited, which is quite different from that reported at 280 nm [20,23,24] where both FQ and Lyz molecules should be excited.

### 2.7. Thermodynamic Parameters and Binding Forces

The interaction forces between a small organic molecule and a biological macromolecule mainly consist of four types: hydrophobic interactions, hydrogen bonding, van der Waals forces and electrostatic interactions. The signs and magnitudes of the thermodynamic parameters (Δ*H* and Δ*S*) can account for the main forces involved in the binding reaction. Ross and Subramanian had pointed out that the signs and magnitudes of the thermodynamic parameters (Δ*H* and Δ*S*) were related with various individual kinds of interaction that may take place in a protein association process, which was summarized as follows: if Δ*H* > 0 and Δ*S* > 0, the main force is hydrophobic interaction. If Δ*H* < 0 and Δ*S* < 0, van der Waals and hydrogen-bonding interactions play major roles in the reaction. Finally, electrostatic forces are more important when Δ*H* < 0 and Δ*S* > 0 [43].

In order to be able to deduce what type of interaction acts between FQs and Lyz, the effect of temperature on the formation of the FQ-Lyz complex was evaluated. Using the van’t Hoff Equation (3) it is possible to obtain both the enthalpy and the entropy of complex formation. The thermodynamic parameters calculated at 298 K at pH 4.5, 7.4 and 10 with the corresponding correlation coefficients (r) are presented in Table 4.

(3) $$lnK=\frac{\Delta S}{R}-\frac{\Delta H}{R}\times \frac{1}{T}$$

where *K* is the binding constant at any particular temperature *T*, *R* is the universal gas constant, Δ*H* is the enthalpy change and Δ*S* is the entropy change of the system.

In all cases, the sign of free energy (Δ*G*) is negative, which indicates that the interaction process between FQ and Lyz is spontaneous. The negatives values of Δ*H* and Δ*S* parameters indicate that the Van der Waals and hydrogen bond interactions are the predominant forces for both FQs at pH 4.5.

At pH 7.4, different interactions are observed for both FQs. In fact, for Cpx, the Van der Waals and hydrogen bond interactions are predominant, while for Lev electrostatic forces are involved. Finally, at pH 10 the negative values of Δ*H* and positive values of Δ*S* for both FQs suggest that the electrostatic forces are predominant in the interaction. Those results are in agreement with the FTIR results mentioned above.

### 2.8. Molecular Docking Studies of the FQs-Lyz Interactions

**Figure 6:**
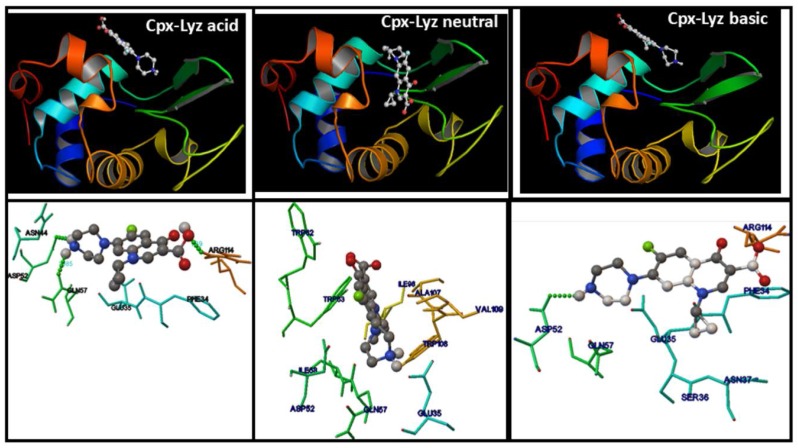
The binding region of Lyz with cationic (pH 4.5), zwitterionic (pH 7.4) and anionic (pH 10) Cpx (**upper**) and amino acid residues around this site (**down**). The hydrogen bond formed with the amino acid residues are in dotted green color.

**Figure 7:**
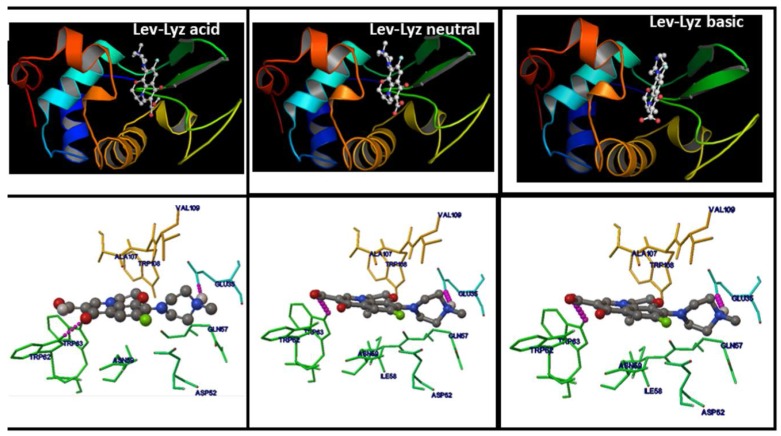
The binding region of Lyz with cationic, zwitterionic and anionic Lev (**upper**) and amino acid residues around this site (**down**). The hydrogen-bond formed with the amino acid residues are in dotted purple color.

For an in depth study of the drug−protein interaction process, the experimental observations were followed up with docking studies, in which the location of the bound drug molecule inside the protein macromolecule and the types of binding forces involved in the FQ-Lyz interactions are explored. The amino acids residues in the binding cavity for Cpx and Lev are shown in Figure 6 and Figure 7, respectively. Similar interactions in the same binding site were observed for Cpx in acid and basic media, where Cpx molecule interact with the α-helix region corresponding to the amide I protein band around of Phe34, Glu35, Ser36, Asn37, Asn44, Asp52, Gln57, Arg114 residues.

In acid medium, we observed the formation of three hydrogen bonds between the amine group and oxygen atom of Cpx molecule and Glu35, Asp52 and Arg114 residues, which present bond lengths of 1.98 Å, 1.77 Å and 1.77 Å, respectively. These analyses are in total agreement with the shift to lower frequencies observed for ν COO^−^ group by infrared spectroscopy and confirm the nature of the interaction mentioned before. In basic medium, a hydrogen bond between anionic Cpx species and the amine group with Asp52 residue is formed with a bond length of 2.186 Å.

Interestingly, the zwitterionic Cpx species, is localized inside the substrate binding site of the protein in the close vicinity of Trp62, Trp63 and Trp108 residues. This site is an amino acid-rich region with few ionic or apolar residues (Ile, Trp, Gln, Ala and Val) including Asp52 and Glu35 also observed for the other media, and they play important roles in the enzymatic activity of Lyz [20]. This interaction confirms the high electronic delocalization in the structure of this species inferred by energies and NBO analysis. In neutral medium, hydrophobic forces without hydrogen bonding guide direction to the entry of Cpx into the protein. Thus, the greater quenching of Cpx fluorescence observed in neutral medium could be attributed to the interaction of the drug with Trp63 and Trp108 located in the binding region inside Lyz.

The analysis of intermolecular energy obtained from docking results can provide information about the binding forces involved. The docking results (Table S3) agree with the experimental results and inferred the nature of FQ-Lyz interaction, with special differences for Cpx and Lyz in neutral medium in comparison with remaining media.

The binding energy obtained by AutoDock simulation, could be assumed as the Gibbs free energy. From this free energy it is possible to calculate de *K_b_* values (binding constant) predicted for Cpx-Lyz interaction (Table S3) which were similar to those obtained experimentally from the fluorescence emission of FQs. We can observe that the main forces involved are the van der Waals contribution energy in acid medium while in basic medium the ionic contribution (electrostatic forces) is greater than the hydrophobic forces and corroborates the previously discussed thermodynamic data obtained from fluorescence assays.

Lev ionic species interact with Lyz protein through two hydrogen bonds in the site around Trp 62 and Glu35 residues according to the predicted ones by docking results. The main nature of the residues in the binding site are mainly hydrophobic and hydrogen bond for the three ionic species of Lev, in spite of what was reported by Fang et al. only in aqueous solution, where they did not determine hydrogen bonds [23].

### 2.9. Effect of the Lyz-FQ Interactions on the Antimicrobial Activity

Taking into account the results of the previous sections that inferred a binding between FQs and Lyz with the formation of a complex we analyze whether this interaction affects the antimicrobial activity of FQs. The assays were carried out pH 4.5 and 7.4 considering that these pH values could be found in the gastrointestinal tract.

FQs are antibiotics that interfere with the maintenance of chromosomal topology by addressing topoisomerases, trapping these enzymes in the DNA cleavage step and preventing the strand rejoining. When they are administrated, FQs can interact with proteins such as Lyz and therefore their antimicrobial activity could be affected. Lyz is a natural protein with bactericidal activity against Gram-positive bacteria, including lactic acid bacteria (LAB). The main antimicrobial target of Lyz is the peptidoglycan of the cell wall. The interaction between two antimicrobial agents have present three main effects on bacterial survival: an additive effect, if the final effect is equal to the sum of the drugs; a synergistic effect, i.e., the effect is greater than the sum and an antagonistic effect (the combination result in less antimicrobial activity compared with each drug alone) [44]. Thus, the effect of Lyz on the antimicrobial action of FQs at different pH values are evaluated.

For these assays we selected a Gram (+) bacterium as a model microorganism. *Lactobacillus (L.) reuteri* CRL 1098 is a LAB with proven probiotic properties [45]. In order to exert its beneficial effect, CRL 1098 strain must remain viable during its passage through the gastrointestinal tract, where FQs could be found during a concomitant treatment. Besides, *L. reuteri* strains are common members of the intestinal microbiota, therefore this study could be useful for a better understanding of the effect of the antibiotics administration on gut bacteria.

**Figure 8:**
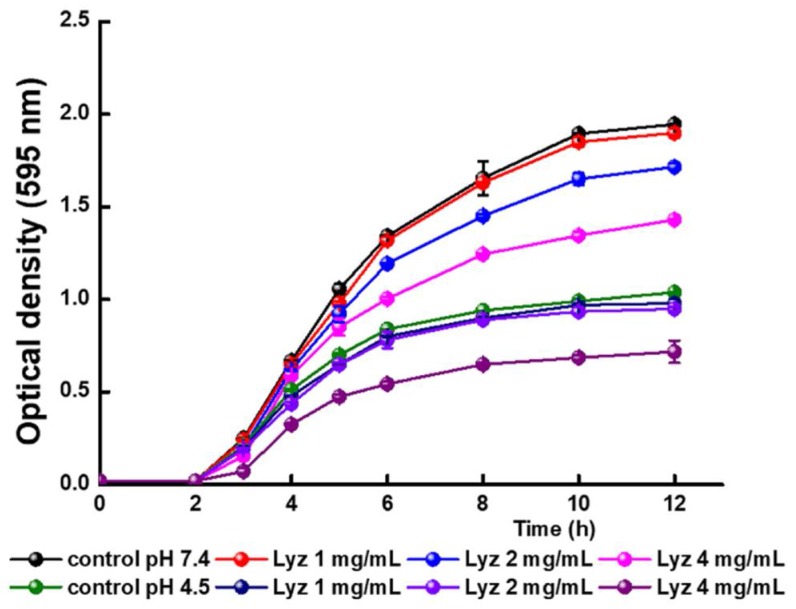
Effect of different concentrations of Lyz on the cell growth of *L. reuteri* CRL1098. Black, red, blue and rose spheres corresponding to assays at pH = 7.4; green, navy, violet and purple corresponding to assays at pH = 4.5.

Figure 8 shows the growth kinetics of *L. reuteri* CRL1098 at different concentrations of Lyz at pH 4.5 and 7.4. The cell viability of the strain is clearly affected by Lyz, the inhibitory effect depending on both the concentration and the pH of the media. In the presence of 1 mg/mL, no inhibitory effect was observed since the same cell concentration as the control is reached at both pH values. At 2 mg/mL Lyz, less than 10% of cell death after 12 h of incubation was observed in the two conditions assayed, while at the highest concentration (4 mg/mL) a strongly inhibition was detected (Figure 8, Table 5). Based on these results, the concentration of 2 mg/mL was employed for further assays.

**Figure 9:**
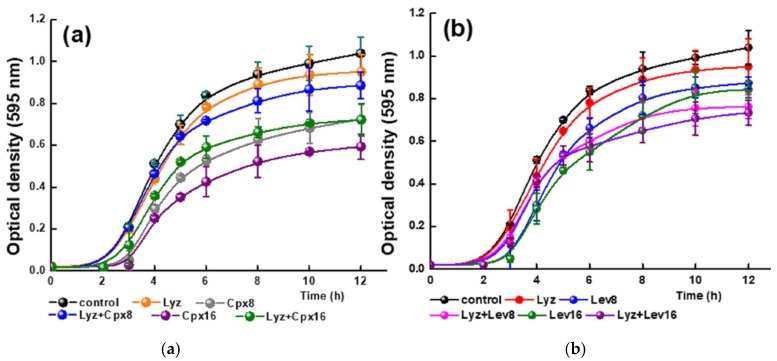
Effect of Cpx (**a**) and Lev (**b**) on the cell growth of *L. reuteri* CRL1098 at pH = 7.4 in the presence and absence of 2 mg/mL Lyz. Cpx8 = Ciprofloxacin 8 μg/mL; Cpx16 = Ciprofloxacin 16 μg/mL; Lev8 = Levofloxacin 8 μg/mL; Cpx16 = Levofloxacin 16 μg/mL.

**Figure 10:**
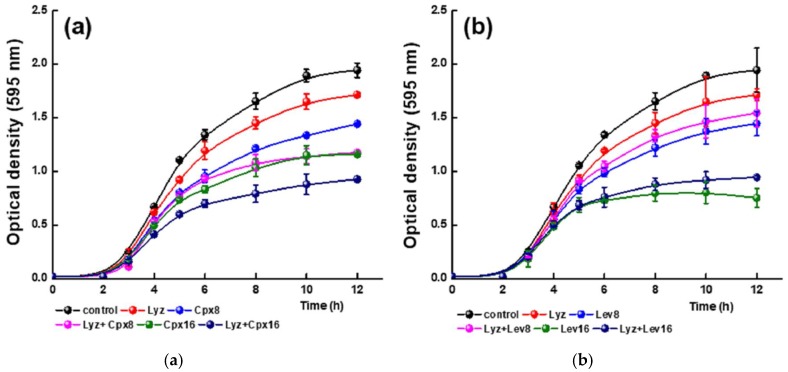
Effect of Cpx (**a**) and Lev (**b**) on the cell growth of *L. reuteri* CRL1098 at pH = 4.5 in the presence and absence of 2 mg/mL Lyz. Cpx8 = Ciprofloxacin 8 µg/mL; Cpx16 = Ciprofloxacin 16 µg/mL; Lev8 = Levofloxacin 8 µg/mL; Cpx16 = Levofloxacin 16 µg/mL.

As shown in Figure 9a and Figure 10a, the antimicrobial activity of Cpx is dependent on the concentration, being slightly higher at pH 7.4. These results are in agreement with those previously reported for FQ since the antibacterial action of Cpx decreased with reductions of pH from 7.4 to 5.5 [46]. At both pH values the lowest concentration evaluated (4 μg/mL) did not affect the cell viability of the CRL 1098 strain (data not shown).

The growth kinetics of *L. reuteri* CRL 1098 at pH 7.4 (Figure 9a) in the presence of Cpx and Lyz differed strongly with respect to the Cpx treatment alone. As is shown in Table 5 at 12 h, 40% of growth inhibition is reached in the presence of Lyz and Cpx (8 μg/mL) value close to the sum of the single treatment, so an additive effect between the protein and Cpx zwitterionic species could be inferred. Similar results are observed at higher concentration of Cpx (16 μg/mL). These results suggest that the interaction between Lyz and Cpx does not substantially modify the activity of the FQ. Previous reports showed that Cpx interacts with DNA (to form the FQ-DNA-topoisomerase complex) with specific hydrogen bonds and van der Waals forces, which involves the COO^−^ group of the Cpx [47]. In neutral medium (Figure 6), the presence of Lyz does not affected the COO^−^ group that could be available to bind with DNA causing cell death, as is described in the molecular docking assays. It is important to highlight at the end of the assay (12 h) a pH of 4.5 is reached (data not shown). However, this change in pH did not seem to modify the described effects. The opposite behavior was observed in acid medium (Figure 10a). Indeed, a combination of both compounds results in an antagonism effect since 14.58% of growth inhibition is observed compared with the 29.96% reached in the absence of Lyz. These results strongly suggest that the complex formation blocks the binding of Cpx with bacterial DNA, inhibiting its antimicrobial activity. This result are in the same direction with those shown in the Figure 6, where hydrogen bond is observed between the COO^−^ group of Cpx and the Lyz.

The presence of Lyz also modified the antimicrobial activity of Lev (Figure 9b and Figure 10b). In fact, at pH 7.4 a moderate antagonistic effect between both compounds was observed during the whole test, being more evident towards the end of the fermentation. At acid pH, a dual effect occurred. Up to 5 h of incubation (the early exponential phase) an antagonistic effect between Lyz and Lev was observed. However, at the end of the assay this behavior was reversed, observing an additive effect between both compounds. These results may suggest that the initial interaction was modified or lost over time. We thus conclude that the Lyz-Cpx complex effectively affects the antimicrobial activity of Cpx at pH 4.5 since a lower inhibitory effect was observed. Due to the Lyz-Lev complex formation a moderate antagonic effect takes place at the two studied pH values.

## 3. Materials and Methods

### 3.1. Materials

Ciprofloxacin (1-cyclopropyl-6-fluoro-4-oxo-7-(piperazin-1-yl)-1,4-dihydroquinoline-3-carboxylic acid) ≥98.0% (HPLC), levofloxacin [(*S*)-9-fluoro-2,3-dihydro-3-methyl-10-(4-methyl-1-piperazinyl)-7-oxo-7*H*-pyrido(1,2,3-de)-1,4-benzoxazine-6-carboxylic acid)] ≥98.0% (HPLC), lysozyme from chicken egg white (lyophilized powder, protein ≥ 90%, ≥40,000 units/mg protein) were purchased from Sigma-Aldrich (St. Louis, MO, USA) and used without further purification. Solid Trizma^®^ base (2-amino-2-hydroxymethyl-propane-1,3-diol) ≥99.9% (titration) was purchased from Fluka (Buchs, Switzerland). Fresh stock solutions of Cpx and Lev (60 mM) were prepared in Trizma base (10 mM). The pH values of 4.5, 7.4, and 10 were adjusted by the addition of HCl and NaOH. The solution stability at each pH was tested for two days and determined using an AD1000 pH meter (Adwa Instruments, Szeged, Hungary). All aqueous solutions were prepared with ultrapure water (conductivity = 0.002–0.010 mS cm^−1^) obtained from an OSMOION 10.2 water purification system (APEMA, Buenos Aires, Argentina).

### 3.2. Computational Calculations 

**Figure 11:**
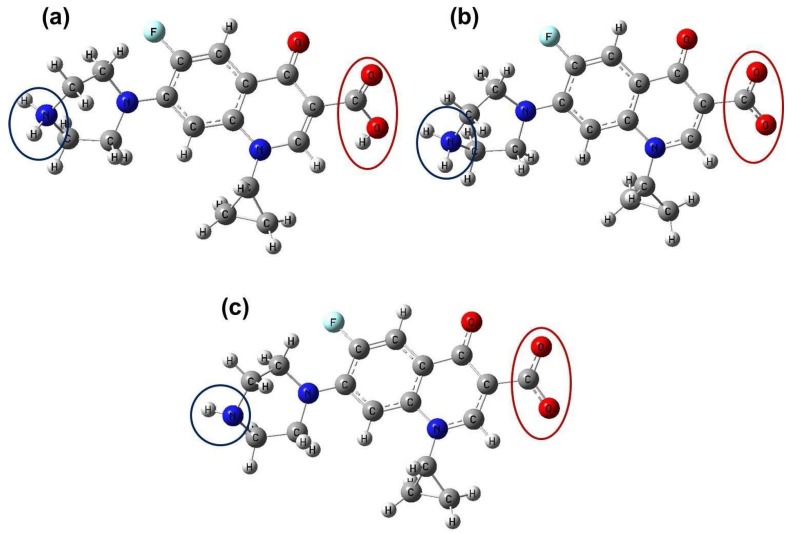
Optimized structures of Cpx molecules in aqueous solution at different pH values: (**a**) 4.5; (**b**) 7.4 and (**c**) 10. Black circles indicate the amine group and red circles the carboxylate group, in each pH medium. The ultraviolet visible (UV-Vis) spectra were also predicted for all species in aqueous solution by using TD-DFT, to evaluate the involved electronic transitions.

The initial structures of each Cpx and Lev species at pH 4.5 (cationic form), 7.4 (zwitterionic form) and 10 (anionic form) were initially built with the GaussView program [48], taking into account the structure of Cpx reported by Zhang et al. [49]. The Cartesian coordinates of these systems were optimized in gas and aqueous solution phases using the hybrid B3LYP/LANL2DZ method with the Gaussian 09 program [50]. The influence of the solvent on Cpx and Lev energies were studied by using the self-consistent reaction field (SCRF) method together with the polarized continuum model (PCM) at the same level of theory [51]. The predicted solvation energies involved in the dissolution process were calculated with the solvation model (PCM/SMD) [52]. The reactivities and behaviors of Cpx and Lev systems in gas phase and aqueous medium were also predicted by using the frontier molecular orbitals. The vibrational spectra of all structures were calculated at the B3LYP/LANL2DZ level of theory that offers the best quality to predict the structure of Cpx, as was reported by Yang et al. [23]. The optimized structures obtained for Cpx at pH 4.5, 7.4 and 10 are shown in Figure 11. The changes in carboxyl and amine groups observed for Cpx were also visualized for the Lev structure.

### 3.3. UV-Visible Measurements

UV-Vis absorption spectra were recorded on an S-2150 Series spectrophotometer (UNICO, Fairfield, NJ, USA) connected to a PC provided with an UNICO Application Software. The measurements were made in 1 cm path quartz cells (Hellma, St. Louis, MO, USA) from 220 nm to 360 nm.

### 3.4. Fluorescence Assays at Different Temperatures

The binding of FQ with Lyz was investigated using fluorescence spectroscopy at three different pH values (4.5, 7.4 and 10), maintaining the samples temperature controlled by a thermostated water bath at 293, 298 and 308 K. All measurements were performed on an SLM 4800 spectrofluorometer, (AMINCO, Urbana, IL, USA) using a 1.0 cm quartz cell in the range of 350–650 nm. The excitation wavelength was 330 nm with a slit of 8 nm. Lysozyme stock solution (20 μM) was dissolved in FQs solutions. FQs samples were prepared with the addition of Lyz (Initial stock solution) at each pH. Different aliquots of FQ-Lyz stock solution were added in FQ solutions in order to obtain different concentrations. Final concentrations of Lyz in the cuvettes were between 1 and 20 μM. Data correspond to an average of at list three experimental runs for each assay.

### 3.5. ATR-FTIR Measurements

ATR-FTIR measurements were recorded in a Thermo Scientific (Waltham, MA, USA) 6700 spectrometer equipped with a DTGS KBr detector and KBr beam splitter. The spectrometer was connected to a system of dry air circulation in order to avoid the interference of water vapor and carbon dioxide. A multiple bounce ATR smart accessory was used to record spectra of Cpx, Lev and Lyz aqueous solution at different pH values with a resolution of 4 cm^−1^, 64 scans and a selected range between 650 and 4000 cm^−1^. Fifty μL of each solution were placed on ZnSe crystal (45°), all spectra were recorded during water evaporation at room temperature and 40% of RH. The FTIR spectra were analyzed using OPUS version 7.0 software (Bruker Optics, Ettlingen, Germany). For secondary structure analysis a combination of Fourier deconvolution and curve-fitting procedures was used to estimates of the position of the component bands and to reconstruct the contours of the original band envelope. Data correspond to an average of at list three experimental runs for each assay.

### 3.6. Molecular Docking

The binding site between Lyz protein with Cpx or Lev at different pH values was studied by molecular docking. All optimized structures of Cpx and Lev antibiotics were used to perform the docking calculation using AutoDock 4.2 too [53] with a semiempirical free-energy force. The crystal structure of Lyz grown at pH 6.5 was obtained from Protein Data Bank (available online: http://www.rcsb.org/pdb, PDB ID: 1F0W) [54] and used for performing the docking calculations at acid and neutral pH, (available online: http://www.rcsb.org/pdb, PDB ID: 1HSX) [55] structure was used for docking in basic medium. All the water molecules present in the macromolecular structure were removed and then, polar hydrogen atoms and Kollman united atom charges were added. Default AutoDock parameters with Lamarckian Genetic Algorithm (LGA) were used (100 runs, the mutation rate of 0.02 and the population size of 200). In order get better the free movement of all structures around the protein, a grid volume (80 × 80 × 80 with 1 Å grid spacing) was considered to evaluate the atomic interactions in the binding sites more precisely. The antibiotics (ligands) and Lyz were treated as rigid docking. The resulting docked conformations were clustered using a root-mean-square deviation (RMSD) tolerance of 1.0 Å. The ligand conformation with the lowest free energy of binding (∆*G*), was chosen from the most favored cluster and was selected for the further analysis.

### 3.7. Effect of Lyz on the Antimicrobial Activity of FQs

#### 3.7.1. Microorganism and Culture Conditions

*Lactobacillus reuteri* CRL 1098, a probiotic strain [45] belonging to CERELA Culture Collection (San Miguel de Tucumán, Argentina) was grown in MRS broth (Merck, Darmstadt, Germany) at 37 °C for 16 h before use. The pH of MRS broth was adjusted to 4.5 and 7.4 (chosen for being pH normally found in the intestinal tract) by adding 5N NaOH or 2N HCl. Solutions of FQs and Lyz were prepared as described above.

#### 3.7.2. Viability Assays

Viability assays were performed in a 96-well microplate. An overnight culture of the lactic strain was inoculated on MRS broth at pH 4.5 or 7.4 supplemented with FQ or Lyz alone or combined. The growth was measured by measuring OD_595nm_ in a microplate reader (Cary 50-UV-VIS spectrophotometer, Varian, Palo Alto, CA, USA) during incubation at 37 °C for 12 h. Lyz was added at a final concentration of 1, 2 or 4 mg/mL, Cpx or Lev at 4, 8 and 16 μg/mL, which was selected according to [45]. In order to evaluate the effect of Lyz on the antimicrobial action of FQs, the following combinations were tested: 2 mg/mL Lyz × 8 μg/mL FQs (Cpx or Lev) and 2 mg/mL Lyz × 16 μg/mL FQs. Cultures at pH 4.5 or 7.4 without the addition of FQs or Lyz were included as controls. The pH was measured using a pH meter (pH 209; Hanna Instruments, Póvoa de Varzim, Portugal). The growth inhibition percentage was calculated by using the following equation: 100 − (Absorbance A/Absorbance B × 100), where A = culture with FQs or Lyz and B = control. All measurements were conducted in at least three independent assays, and the obtained results were reported as mean values with their respective standard deviations. The analysis of variance (ANOVA) and Fisher’s least significant difference (LSD) post-hoc tests were performed using InfoStat. Grupo InfoStat, FCA, Universidad Nacional de Córdoba, Argentina, Available online: http/www.infostat.com.ar. All statistical analyses were conducted at a significance level of *p* < 0.05. Data correspond to an average of at list three experimental runs for each assay.

## 4. Conclusions

A detailed study on the interaction of fluoroquinolones with lysozyme at different pH conditions was conducted. Using theoretical calculations, a high ΔG_solv_ for the Cpx zwitterionic species present at neutral pH is observed, indicating that the dissolution reaction occurs spontaneously. The aqueous medium confer more stabilization energy for the zwitterionic species especially for the Lev antibiotic. Furthermore, frontier orbitals and Natural Bond Orbital analyses confirm and show that the two charged centers (NH_2_^+^ and COO^−^) increase their stability by electronic delocalization. These results evidently show that the thermodynamic factor is the major responsible of the stability of the stability of FQ in aqueous solution.

Using fluorescence and FTIR spectroscopy we demonstrated that the zwitterionic Cpx species binds more strongly to lysozyme than the cationic one and slightly alter its α-helix structure, while in basic medium the Cpx-Lyz binding favor an increase of the α-helix structure. At acid and neutral pH, the binding of Cpx and Lev caused a quenching that indicates that FQ bind to lysozyme via a static quenching mechanism.

For Cpx, hydrogen bonding and Van der Waals forces are mainly responsible for the interactions in acid and neutral media, while electrostatic forces drive them in basic medium. We also demonstrated that the amine and carboxylate groups are blocked by hydrogen bonding with the Asp and Glu residues of Lyz. In neutral medium the hydrophobic residues such as Trp, Val, Ile are responsible for the interaction with the protein, but the amine and carboxyl group are available for other molecules. For Lev, the interaction mode is independent of the pH and the hydrogen bond is the main force involved, in agreement with DFT results.

Antimicrobial assays confirmed the docking results. In fact, the antagonist effect between Cpx and Lyz observed in acid medium would prove that the protein blocks the oxygen atoms and amine group of the Cpx, preventing its binding to DNA and therefore modifying its antimicrobial activity. On the other hand, due to the Lyz-Lev complex formation a moderate antagonic effect take place at the two studied pHs. The findings analyzed in this work provide valuable information to better understand the interaction mechanism of FQs with Lyz at different active sites promoted by the solvent medium. Further studies will be useful for a new approach to the interaction site of these antibiotics in their biological environment and could eventually lead complexes formation.

## Figures and Tables

**Table 1:** Calculated (B3LYP/LAN2DZ) stabilization energy (E) relative energies (ΔE) and free energy solvation (ΔG_solv_), dipolar moment (μ), for Cpx and Lev in aqueous solution at different pH values.

Ciprofloxacin				
pH	E	ΔE (kJ/mol)	ΔG_solv_ (kJ/mol)	μ(D)
4.5	−1148.74	0	−385.9	42.2
7.4	−1148.28	1206	−440.7	61.9
10	−1147.81	2413	−348.9	36.2
**Levofloxacin**				
4.5	−1261.97	0	−426.9	31.7
7.4	−1261.51	263.4	−515.5	58.6
10	−1261.04	558.2	−418.7	33.9

**Table 2:** Percentage of secondary structure (amide I) present in the structure of Lyz by interaction with Cpx and Lev at different pH values.

	LYSOZYME						
	1606 cm^−1^	1623 cm^−1^	1630 cm^−1^	1640 cm^−1^	1652 cm^−1^	1670 cm^−1^	1690 cm^−1^
pH	Extended	Parallel β-Sheet	Antiparallel β-Sheet	Unordered Coil	α-Helix	Turn	Parallel β-Sheet
4.5	15 ± 3		12 ± 1	24 ± 4	29 ± 1	20 ± 1	10 ± 1
7.4	10 ± 2		5 ± 1	30 ± 2	31 ± 3	24 ± 1	
10	8 ± 1	9 ± 2	11 ± 2	23 ± 3	21 ± 2	18 ± 1	
	**LYSOZYME—CIPROFLOXACIN**						
4.5	15 ± 1		17 ± 2	22 ± 1	28 ± 2	18 ± 1	
7.4	18 ± 1		14 ± 1	20 ± 3	29 ± 2	19 ± 3	
10	10 ± 3	6 ± 1	11 ± 2	20 ± 1	27 ± 1	18 ± 1	8 ± 1
	**LYSOZYME—LEVOFLOXACIN**						
4.5	21 ± 2		17 ± 1	16 ± 3	29 ± 2	17 ± 1	
7.4	18 ± 2		18 ± 3	19 ± 3	26 ± 1	19 ± 1	
10	18 ± 1	10 ± 1	7 ± 2	18 ± 2	25 ± 2	14 ± 2	8 ± 2

**Table 3:** Binding constants for the interaction of FQs with Lyz at different temperatures.

		pH = 4.5			pH = 7.4			pH = 10		
		*K* (10^4^ L mol^−1^)	SD *	r **	*K* (10^4^ L mol^−1^)	SD *	r **	*K* (10^4^ L mol^−1^)	SD *	r **
Lyz—Cpx	298 K	0.41	0.01	0.997	1.80	0.01	0.988	4.05	0.01	0.994
	308 K	0.31	0.02	0.983	1.13	0.02	0.992	3.24	0.02	0.995
Lyz—Lev	298 K	0.97	0.02	0.988	0.27	0.02	0.991	15.15	0.02	0.994
	308 K	0.69	0.10	0.982	0.22	0.10	0.989	13.93	0.10	0.987

* SD standard deviation, and ** r correlation coefficient.

**Table 4:** Binding and thermodynamic parameters of the Cpx—Lyz at different pH values.

	pH	Δ*H* (kJ mol^−1^)	Δ*S* (J mol^−1^K^−1^)	Δ*G* (kJ mol^−1^)	r
**Cpx-Lyz**	*4.5*	−24.04 ± 0.90	−11.32 ± 2.93	−20.64 ± 0.10	0.997
	*7.4*	−37.55 ± 1.73	−44.46 ± 5.78	−24.27 ± 0.22	0.996
	*10*	−15.53 ± 1.26	35.95 ± 4.21	−26.28 ± 0.26	0.987
**Lev-Lyz**	*4.5*	−26.27 ± 1.14	−11.82 ± 1.12	−22.76 ± 0.07	0.989
	*7.4*	−14.98 ± 0.84	15.32 ± 2.45	−19.57 ± 0.15	0.993
	*10*	−6.39 ± 1.22	77.70 ± 4.13	−29.55 ± 0.31	0.998

**Table 5:** Effect of Lyz and FQs on the survival of *L. reuteri* CRL 1098.

	Inhibition of Growth (%)	
Treatments	pH = 4.5	pH = 7.5
Lyz 2 mg/mL	8.65 ± 0.7 ^a^	18.70 ± 1.1 ^a^
Cpx 8 μg/mL	29.96 ± 1.5 ^b^	25.84 ± 1.7 ^b^
Cpx 8 μg/mL + Lyz	14.58 ± 0.9 ^c^	39.57 ± 1.3 ^c^
Cpx 16 μg/mL	42.83 ± 2.2 ^d^	40.33 ± 2.3 ^c^
Cpx 16 μg/mL + Lyz	30.22 ± 1.6 ^b^	52.32 ± 3.3 ^d^
Lev 8 μg/mL	15.99 ± 0.9 ^c^	25.69 ± 1.5 ^b^
Lev 8 μg/mL + Lyz	26.54 ± 2.5 ^e^	20.50 ± 2.2 ^d^
Lev 16 μg/mL	18.93 ± 1.7 ^f^	61.30 ± 5.8 ^e^
Lev 16 μg/mL + Lyz	29.36 ± 3.5 ^b^	51.40 ± 4.8 ^d^

Variables with the same letter in superscript in the same column are not significantly different (*p* < 0.05).

## Supplementary Materials

The following are available online, Figure S1: HOMO and LUMO molecular orbitals by Cpx species in basic medium; Table S1: Calculated HOMO and LUMO orbitals Energies, band gap (GAP) and chemical potential (μ) for two antibiotic molecules in aqueous solution; Table S2: Main donor-acceptor energy interactions (in kJ/mol) for all FQ species by using the hybrid B3LYP level of theory and the Lanl2dz basis sets, Table S3: Binding constant (*K_b_*), free energy (ΔG), electrostatic interaction energy (E) and van der Waals, hydrogen bonding interaction energies (Evdw) obtained from docking results.
