# Supplementary material for: Effects of Lysozyme on the Activity of Ionic of Fluoroquinolone Species
Source: Molecules. 2018 Mar 23;23(4):741. doi: 10.3390/molecules23040741 (PMC6017444; doi:10.3390/molecules23040741)
Supplement: Supplementary file 1 [file molecules-23-00741-s001.pdf]

Supporting Information

Lysozyme Affects the Activity of Fluoroquinolones Species at Different pHs

Hugo Alejandro Perez [1,2], Ana Yanina Bustos [3,4], Maria Pia Taranto[5], María de los Angeles Frías [2] and Ana Estela Ledesma [1]

[1] Departamento de Química, Facultad de Ciencias Exactas y Tecnologías, Universidad Nacional de Santiago del Estero-CONICET, Av. Belgrano (S) Nº 1912, 4200, Santiago del Estero, Argentina.
[2] Laboratorio de Biointerfases y Sistemas Biomimeticos, Centro de Investigación en Biofísica Aplicada y Alimentos (CIBAAL) Universidad Nacional de Santiago del Estero - CONICET, RN 9- Km 1125, 4206, Santiago del Estero, Argentina.
[3] Centro de Investigación en Biofísica Aplicada y Alimentos (CIBAAL) Universidad Nacional de Santiago del Estero - CONICET, RN 9- Km 1125, 4206, Santiago del Estero, Argentina.
[4] Facultad de Ciencias Médicas, Universidad Nacional de Santiago del Estero-CONICET, Av. Belgrano (S) Nº 1912, 4200, Santiago del Estero, Argentina.
[5] Centro de Referencia para Lactobacilos (CERELA-CONICET), Chacabuco 145, 4000, San Miguel de Tucumán, Argentina.

* Correspondence: e-mail: ana1ledesma@yahoo.com.ar; Tel.: +543854509560

Table S1. Calculated HOMO and LUMO orbitals Energies, band gap (GAP) and chemical potential (μ) for two antibiotic molecules in aqueous solution.

**Orbitals (eV)**	**pH acid**		**pH neutral**		**pH basic**	
	**Cpx**	**Lev**	**Cpx**	**Lev**	**Cpx**	**Lev**
**HOMO**	-6.1776	-6.3952	-5.6573	-5.7775	-5.5564	-5.5433
**LUMO**	-1.9978	-2.6011	-1.5479	-2.2040	-1.4388	-2.2774
**GAP**	**-4.1797**	**-3.7941**	**-4.1100**	**-3.3754**	**-4.1100**	**-3.2658**
μ	**-4.0877**	**-4.4981**	**-3.6026**	**-3.9907**	**-3.4976**	**-3.9103**

Table S1 shows the highest occupied molecular orbital (HOMO), the lowest unoccupied molecular orbital (LUMO) energies and GAP values calculated at B3LYP/LANL2DZ level of theory and chemical potential (μ) for each FQs species. HOMO molecular frontier orbitals give the most nucleophilic site. As it was reported, when GAP is low, the molecule has high chemical reactivity and low kinetic stability [40]. The difference energy between HOMO and LUMO is defined as gap energy (GAP) and their value determines the chemical reactivity and kinetic stability of a molecule [40].

Table S2. Main donor-acceptor energy interactions (in kJ/mol) for all FQs species by using the hybrid B3LYP level of theory and the Lanl2dz basis sets

**Delocalization**	**CPx**		
	**acid**	**zwiterionic**	**basic**
*π C15-C16 →π*C17-C19*	14.45	14.02	13.90
*π C15-C16 →π*C18-C20*	21.86	19.08	22.16
*π C17-C19 →π*C15-C16*	22.51	22.27	21.38
*π C17-C19 →π*C18-C20*	15.62	14.20	14.02
*π C18-C20 →π*C15-C16*	21.37	23.79	20.30
*π C18-C20 →π*C17-C19*	21.22	22.75	22.59
*π C18-C20 →π*C22-O28*	20.88	19.87	21.98
*π C23-C24 →π*C22-O28*	27.55	27.00	26.58
*π C23-C24 →π*C29-O31*	26.37	0.63	13.94
$\Delta ET_{\pi\rightarrow\pi^*}$	191.83	163.61	176.85
*LP(1)N3 →π*C15-C16*	34.92	29.98	40.71
*LP(3)F26 →π*C17-C19*	12.91	12.01	12.40
*LP(1)N27 →π*C18-C20*	37.29	40.28	39.45
*LP(1)N27 →π*C23-C24*	44.59	32.87	33.82
*LP(2)O30 →π*C29-O31*	47.54		
$\Delta ET_{LP\rightarrow\pi^*}$	177.25	115.14	126.38
*LP(2)O28 →σ*C20-C22*	15.21	15.01	14.48
*LP(2)O28 →σ*C22-C24*	16.41	15.03	15.13
*LP(2)O31 →σ*C24-C29*	15.55		15.30
*LP(2)O31 →σ*C29-O30*	28.06	17.60	17.40
*LP(1)C43 →σ*C16-O44*	18.31	18.38	
$\Delta ET_{LP\rightarrow\sigma^*}$	93.54	66.02	62.31
LP(2)O30 → LP(1)C29		246.48	
LP(2)O31 → LP(1)C29		213.51	
LP(2)O44 → LP(2)C43	71.86	70.58	74.82
$\Delta ET_{LP\rightarrow LP}$	71.86	530.57	74.82
*LP(3)O31 →π*C29-O30*			103.21
*LP(2)O44 → π*C15-C16*	16.84	17.10	15.84
$\Delta ET_{LP\rightarrow\pi^*}$	16.84	17.10	119.05
*π*C15-C16 →π*C17-C19*	85.02	84.31	106.83
*π*C15-C16 →π*C18-C20*	119.72	88.22	116.78
*π*C18-C20 →π*C22-O28*		200.55	
*π*C22-O28 →π*C23-C24*		64.90	72.23
*π*C23-C24 →π*C29-O30*			50.11
*π*C29-O31 →π*C23-C24*	127.73		
$\Delta ET_{\pi^*\rightarrow\pi^*}$	332.47	437.98	345.95
ΔE_{Total}	**883.79**	**1,330.42**	**950.36**

Three different interactions $\Delta ET_{\pi\rightarrow\pi^*}$,$\Delta ET_{LP\rightarrow\pi^*}$, $\Delta ET_{LP\rightarrow\sigma^*}$, and $\Delta ET_{\pi^*\rightarrow\pi^*}$ were predicted, being the $\Delta ET_{\pi^*\rightarrow\pi^*}$ delocalization the ones that present the higher contribution in energy

Table S3. Binding constant (k_b), Free Energy (ΔG), Electrostatic interaction Energy (E) and van der Waals, hydrogen bonding interaction energy (Evdw) obtained from docking results

pH	K_b (mol^{-1} L)	ΔG° (kJ $mol^{-1}K^{-1}$)	E (kJ/mol)	Evdw (kJ/mol)
		**Cpx-Lyz**		
4.5	7.7x 10^4	-27.68 (-20)	-11.54	-21.11
7.5	5.49 x10^5	-32.75 (-25)	- 6.31	-30.14
10	6.66 x 10^4	-27.50 ?	-10.32	-20.9
		**Lev-Lyz**		
4.5	1.24 x10^5	-29.05	-5.39	-27.42
7.5	2.52 x10^5	-30.81	-5.43	-27.88
10	2.49 x10^5	-30.76	-4.85	-28.42

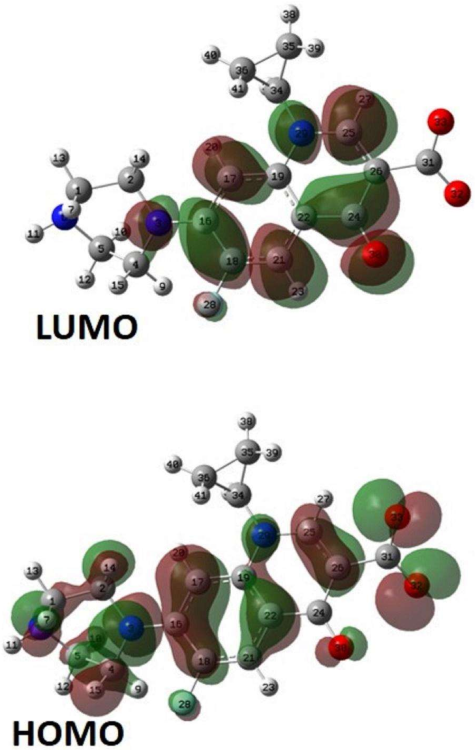

Figure S1.. HOMO and LUMO molecular orbitals by Cpx specie in basic medium

Figure S1 illustrates electron density of the frontier orbitals (contours surface). A uniform electron density over the piperazine and quinolone rings including the carboxylate group in HOMO orbitals (H) with bonding characters was observed. LUMO orbitals (L) are expanded over the atoms of the quinolone ring excluding the carboxylate group and indicating a high antibonding nature.
